# Supplementary material for: Splice-Junction-Based Mapping of Alternative Isoforms in the Human Proteome
Source: Cell Rep. Author manuscript; Available in PMC 2020 Jan 15. (PMC6961840; doi:10.1016/j.celrep.2019.11.026)
Supplement: 2 [file NIHMS1546469-supplement-2.zip › DF1/PXD010154/Thyroid/Thyroid_33_TENM3_IFPSGNVTSVLELR.pdf]

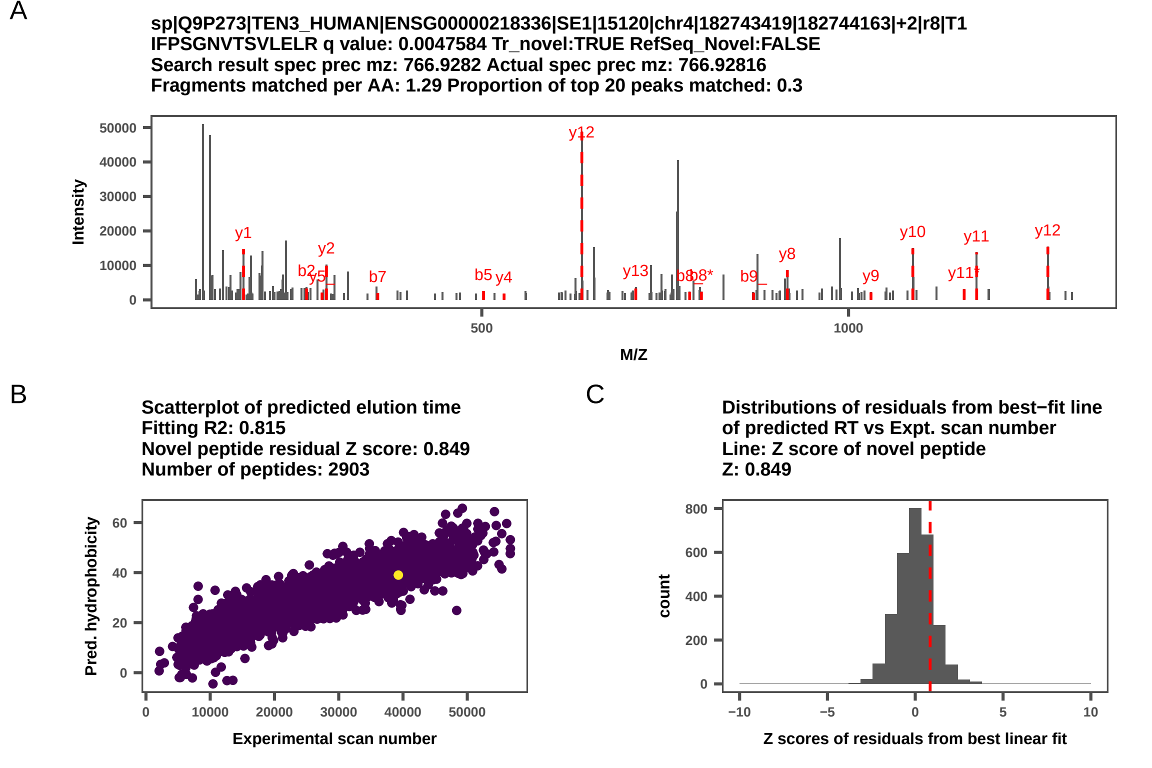
A
sp|Q9P273|TEN3_HUMAN|ENSG00000218336|SE1|15120|chr4|182743419|182744163|+2|r8|T1
IFPSGNVTSVLELR q value: 0.0047584 Tr_novel:TRUE RefSeq_Novel:FALSE
Search result spec prec mz: 766.9282 Actual spec prec mz: 766.92816
Fragments matched per AA: 1.29 Proportion of top 20 peaks matched: 0.3
Intensity
50000
40000
30000
20000
10000
0
y1
y2
b2
y5
b7
b5
y4
y12
y13
b8
b8*
b9
y8
y9
y10
y11
y11*
y12
500
1000
M/Z
B
Scatterplot of predicted elution time
Fitting R2: 0.815
Novel peptide residual Z score: 0.849
Number of peptides: 2903
Pred. hydrophobicity
60
40
20
0
0
10000
20000
30000
40000
50000
Experimental scan number
C
Distributions of residuals from best−fit line
of predicted RT vs Expt. scan number
Line: Z score of novel peptide
Z: 0.849
count
800
600
400
200
0
−10
−5
0
5
10
Z scores of residuals from best linear fit
